# Supplementary material for: The Epidemiological, Clinical, and Microbiological Features of Patients with Burkholderia pseudomallei Bacteraemia—Implications for Clinical Management
Source: Trop Med Infect Dis. 2023 Oct 24;8(11):481. doi: 10.3390/tropicalmed8110481 (PMC10675116; doi:10.3390/tropicalmed8110481)
Supplement: Supplementary file 1 [file tropicalmed-08-00481-s001.zip › tropicalmed-2594738-supplementary.pdf]

Supplementary Table S1. Correlation matrix showing associations between variables.

	Male	Immunosuppression	Wet season presentation	SSTI	No risk factors	Age	First Nations Australian	Remote residence	Diabetes mellitus	Hazardous alcohol use	Renal disease	Chronic lung disease	Malignancy	Lung involvement	GU involvement	MS involvement	CNS involvement
Male	1.00	-	-	-	-	-	-	-	-	-	-	-	-	-	-	-	-
Immunosuppression	0.01	1.00	-	-	-	-	-	-	-	-	-	-	-	-	-	-	-
Wet season presentation	0.01	0.04	1.00	-	-	-	-	-	-	-	-	-	-	-	-	-	-
SSTI	-0.12	-0.10	-0.10	1.00	-	-	-	-	-	-	-	-	-	-	-	-	-
No risk factors	-0.05	-0.17	0.05	0.21	1.00	-	-	-	-	-	-	-	-	-	-	-	-
Age	0.10	0.10	-0.05	-0.22	-0.28	1.00	-	-	-	-	-	-	-	-	-	-	-
First Nations Australian	-0.07	-0.10	-0.02	0.01	-0.14	-0.39	1.00	-	-	-	-	-	-	-	-	-	-
Remote residence	0.10	-0.04	0.06	0.05	-0.12	-0.14	0.33	1.00	-	-	-	-	-	-	-	-	-
Diabetes mellitus	0.01	-0.20	-0.08	-0.06	-0.33	0.01	0.33	0.15	1.00	-	-	-	-	-	-	-	-
Hazardous alcohol use	0.18	-0.06	-0.06	-0.06	-0.25	-0.05	0.02	0.12	-0.13	1.00	-	-	-	-	-	-	-
Renal disease	0.07	-0.06	-0.06	-0.03	-0.13	0.31	0.19	0.02	0.17	-0.02	1.00	-	-	-	-	-	-
Chronic lung disease	0.01	0.04	0.00	-0.09	-0.21	0.23	-0.19	-0.14	-0.19	-0.04	-0.02	1.00	-	-	-	-	-
Malignancy	0.05	0.44	0.02	-0.12	-0.15	0.23	-0.19	0.09	-0.18	-0.10	0.03	0.12	1.00	-	-	-	-
Lung involvement	0.13	-0.02	0.17	-0.33	-0.20	0.18	0.02	0.05	0.01	0.06	0.03	0.19	-0.03	1.00	-	-	-
GU involvement	0.19	-0.10	-0.05	-0.11	-0.05	-0.02	-0.09	0.05	0.18	0.18	0.04	0.02	-0.08	0.01	1.00	-	-
MS involvement	0.04	0.08	-0.03	0.26	-0.04	-0.03	0.07	0.08	0.16	-0.01	-0.04	-0.01	-0.07	-0.03	--0.03	1.00	-
CNS involvement	0.01	-0.08	-0.01	-0.02	0.10	-0.02	-0.11	0.02	-0.09	0.03	-0.01	-0.02	-0.07	0.05	-0.09	-0.01	1.00

SSTI: Skin or soft tissue infection; GU: Genitourinary; MS: musculoskeletal; CNS: Central nervous system
